# KMT5A downregulation participated in High Glucose-mediated EndMT via Upregulation of ENO1 Expression in Diabetic Nephropathy

**DOI:** 10.7150/ijbs.62867

**Published:** 2021-10-03

**Authors:** Lihong Lu, Xue Li, Ziwen Zhong, Wenchang Zhou, Di Zhou, Minmin Zhu, Changhong Miao

**Affiliations:** 1 Department of Anesthesiology, Fudan University Shanghai Cancer Center; Department of Oncology, Shanghai Medical College, Fudan University, Shanghai 200032, China.; 2 Department of Anesthesiology, Zhongshan Hospital, Fudan University, Shanghai, 200032, China.; 3 Department of Anesthesiology, Affiliated Hospital of North Sichuan Medical College, Nanchong, China.; 4 Department of Anesthesiology, Shanghai General Hospital, Shanghai Jiao Tong University School of Medicine, Shanghai 200080, People's Republic of China.

**Keywords:** hyperglycemia, endothelial, diabetic nephropathy, KMT5A

## Abstract

Diabetic nephropathy (DN) has become the common and principal microvascular complication of diabetes that could lead to end-stage renal disease. It was reported endothelial-to-mesenchymal transition (EndMT) in glomeruli plays an important role in DN. Enolase1 (ENO1) and Lysine Methyltransferase 5A (KMT5A) were found to modulate epithelial-to-mesenchymal transition in some situations. In the present study, we speculated KMT5A regulates ENO1 transcript, thus participating in hyperglycemia-induced EndMT in glomeruli of DN. Our study represented vimentin, αSMA and ENO1 expression elevated, and CD31 expression decreased in glomeruli of DN participants and rats. *In vitro*, high glucose induced EndMT by increase of ENO1 levels. Moreover, high glucose downregulated KMT5A levels and increased regulatory factor X1 (RFX1) levels. KMT5A upregulation or si-RFX1 decreased high glucose-induced ENO1 expression and EndMT. RFX1 overexpression- or sh-KMT5A-induced EndMT was attenuated by si-ENO1. Further, the association between KMT5A and RFX1 was verified. Furthermore, histone H4 lysine20 methylation (the direct target of KMT5A) and RFX1 positioned on ENO1 promoter region. sh-KMT5A enhanced positive action of RFX1 on ENO1 promoter activity. KMT5A reduction and RFX1 upregulation were verified in glomeruli of DN patients and rats. KMT5A associated with RFX1 to modulate ENO1, thus involved in hyperglycemia-mediated EndMT in glomeruli of DN.

## Introduction

Diabetic nephropathy (DN) has become the common and principal microvascular complication of diabetes, that could lead to end-stage renal disease 1, 2. The characteristics of DN includes urine albumin increasing, blood pressure elevating, and glomerular filtration rate reducing 3, 4. So far, treatment approaches for DN are very limited. Once progression to end-stage renal disease, the cardiovascular-related mortality augmented, as well as the cost of treatment 5, 6. So, studies for the underlying mechanisms of DN is urgent and important. It has been reported that endothelial-to-mesenchymal transition (EndMT) in glomeruli takes an important part in the genesis and development of DN 7, 8.

EndMT is characterized by drop of the endothelial phenotype including CD31 and obtainment of mesenchymal feature including α-smooth muscle actin (αSMA) and vimentin in endothelial cells 9. EndMT has been reported as a source of collagen-producing myofibroblasts responsible for fibrosis and progression of DN 10, 11. Moreover, inhibition of EndMT alleviated collagen production and fibrosis 12. As a part of epithelial-to-mesenchymal transition (EMT), the modulators of EMT may also take a crucial part in modulation of EndMT 13. Enolase1 (ENO1) plays a vital role in aerobic glycolysis via transformation of 2-phosphoglycerate into phosphoenolpyruvate 14, 15. Moreover, ENO1 was reported to participate in hyperglycemia-induced EMT 16. However, whether ENO1 is also involved in hyperglycemia-induced EndMT is still not studied.

Lysine Methyltransferase 5A (KMT5A), the only confirmed histone lysine methyltransferase, participates in the regulation of the methylation levels of histone H4 lysine 20 (H4K20me1) 17. Previous study has indicated that KMT5A modulates angiogenesis in human umbilical vein endothelial cells (HUVECs) 18. Our previous research illustrated KMT5A is involved in hyperglycemia-induced endothelial damage 19-23. Moreover, KMT5A takes part in the modulation of EMT 24. However, the underlying mechanism by which KMT5A participates in hyperglycemia-mediated EndMT is still not well studied. In this study, we speculate that KMT5A may regulate ENO1 levels, thus participating in hyperglycemia-induced EndMT in vascular endothelium. More importantly, we probed the underlying mechanism by which KMT5A regulates ENO1 levels.

## Results

### Decrease of CD31 levels, augment of vimentin and αSMA expression, and increase of ENO1 levels in DN patients and rats

**Figure 1:**
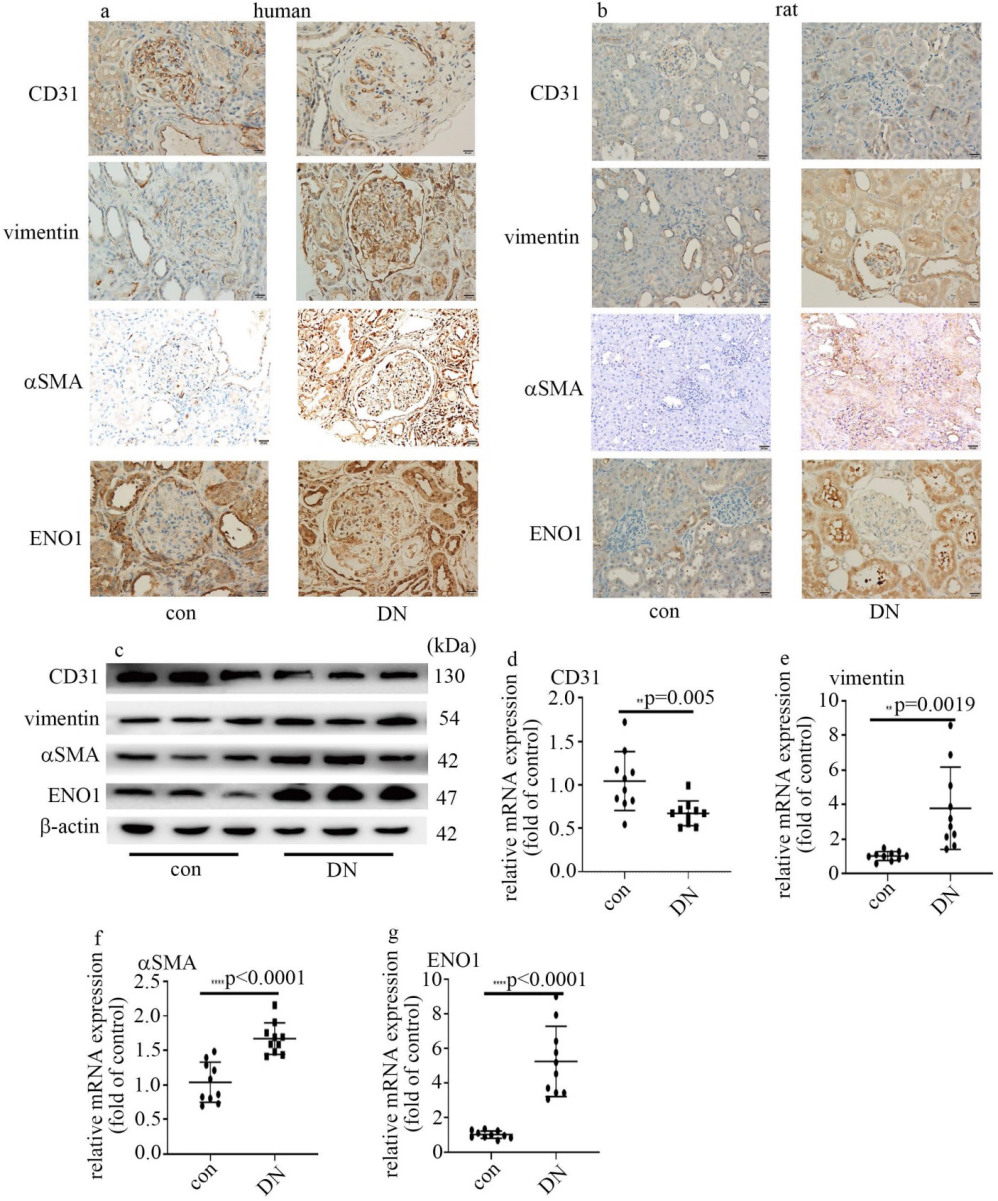
** Decrease of CD31 levels, augment of vimentin and αSMA expression, and increase of ENO1 expression in DN patients and rats. (a)** IHC results of CD31, vimentin, αSMA and ENO1 in kidney tissue of control and DN participates (n = 20/group). Magnification: ×20. Scale bar: 20 μM. **(b)** IHC results of CD31, vimentin, αSMA and ENO1 in kidney tissue of control and DN rats (n = 10/group). Magnification: ×20. Scale bar: 20 μM. **(c)** Protein levels of CD31, vimentin, αSMA and ENO1 in kidney tissue of control and DN rats (n = 10/group). **(d)** mRNA levels of CD31 were tested by qPCR in kidney tissue of control and DN rats (n = 10/group). **(e)** mRNA levels of vimentin were tested by qPCR in kidney tissue of control and DN rats (n = 10/group). **(f)** mRNA levels of αSMA were tested by qPCR in kidney tissue of control and DN rats (n = 10/group). **(g)** mRNA levels of ENO1 were tested by qPCR in kidney tissue of control and DN rats (n = 10/group). (* p<0.05, ** p<0.01, *** p<0.001, **** p<0.0001).

The information of the participants enrolled in this study was listed in Table 1. It has been reported that EndMT in glomeruli takes a crucial part in DN 7, 8. So, we probed CD31, vimentin and αSMA in kidney tissue of control and DN participates. Our data represented CD31 levels reduced, and vimentin and αSMA levels augmented in glomeruli of DN patients (Figure 1a). It was reported that ENO1 participated in hyperglycemia-induced EMT 13. So, we probed ENO1 levels, and found ENO1 levels in glomeruli augmented in DN patients (Figure 1a).

The information of the rats allocated in the present study was shown in Table 1. Similarly, the protein and mRNA expression of vimentin, αSMA and ENO1 in kidney tissue (Figure 1b, c, e-g) and aorta tissues (Figure S1a, b, d-f) of DN rats upregulated, compared with those of the control group. The expression of CD31 in kidney tissue (Figure 1b-d) and aorta tissues (Figure S1a-c) of DN rats downregulated, compared with those of the control group. Our results may represent that EndMT and ENO1 was involved in the genesis and development of DN.

### High glucose mediated EndMT via the increase of ENO1 levels in HUVECs

**Figure 2:**
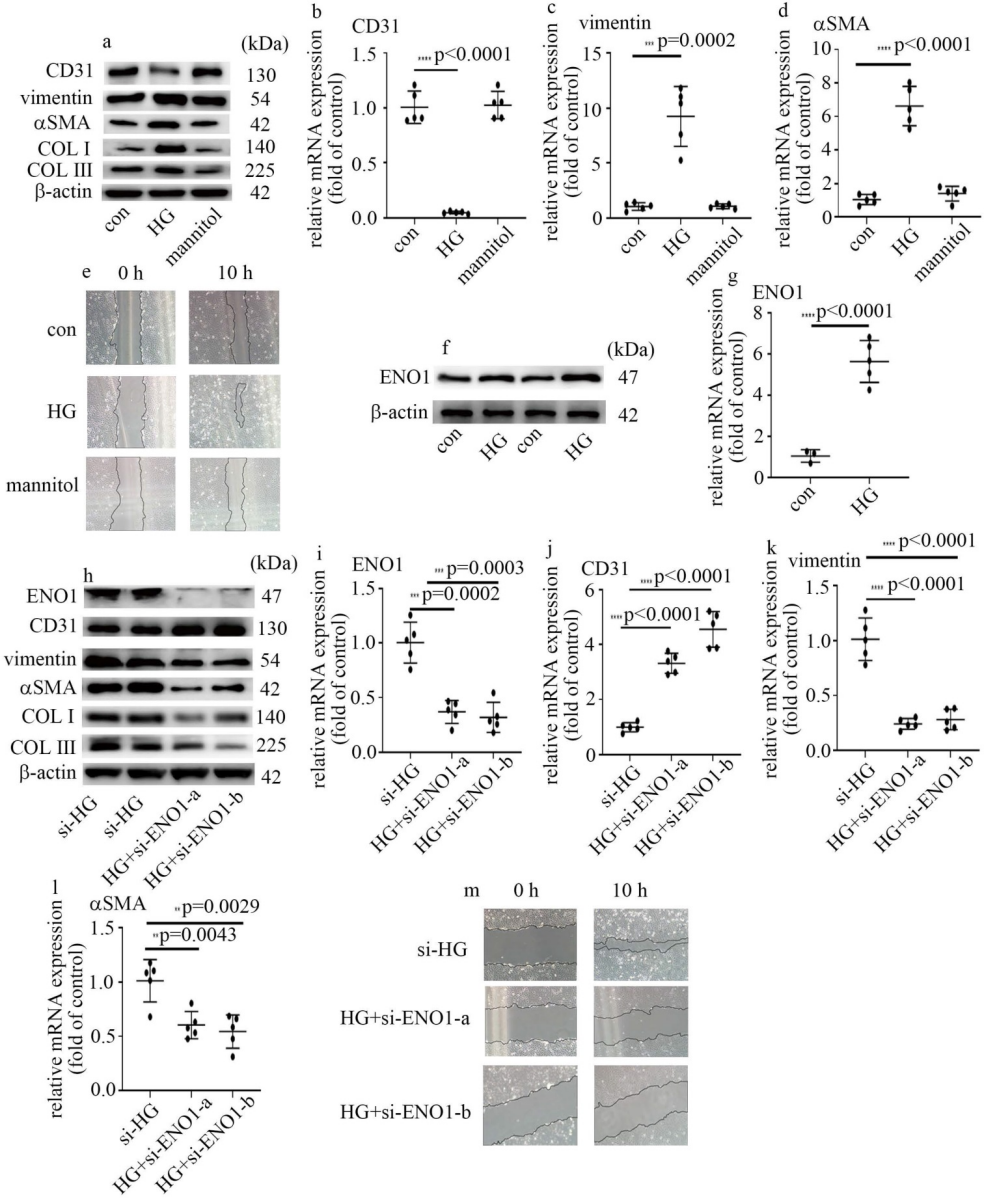
** High glucose mediated EndMT via the increase of ENO1 levels in HUVECs. (a)** Protein levels of CD31, vimentin, αSMA, COLI and COLIII were tested by western blot in HUVECs with corresponding treatment. **(b)** mRNA levels of CD31 were tested by qPCR with corresponding treatment**. (c)** mRNA levels of vimentin were tested by qPCR with corresponding treatment. **(d)** mRNA levels of αSMA were tested by qPCR with corresponding treatment. **(e)** Cell migration was detected by scratch test. **(f)** Protein levels of ENO1 were tested by western blot in HUVECs with corresponding treatment. **(g)** mRNA levels of ENO1 were tested by qPCR with corresponding treatment. **(h)** Protein levels of ENO1, CD31, vimentin, αSMA, COLI and COLIII were tested by western blot in HUVECs with corresponding treatment. **(i)** mRNA levels of ENO1 were tested by qPCR with corresponding treatment. **(j)** mRNA levels of CD31 were tested by qPCR with corresponding treatment. **(k)** mRNA levels of vimentin were tested by qPCR with corresponding treatment. **(l)** mRNA levels of αSMA were tested by qPCR with corresponding treatment. **(m)** Cell migration was detected by scratch test. (* p<0.05, ** p<0.01, *** p<0.001, **** p<0.0001, n=5/group).

To probe the underlying mechanism by which high glucose induced EndMT in glomeruli of DN participants and rats, HUVECs were used in this research. To explore whether hyperglycemia was involved in the occurrence of EndMT and increase of ENO1 levels, cells cultured in DMEM with high glucose were employed as an *in vitro* model. Our data represented high glucose decreased CD31 levels, and augmented vimentin, αSMA, collagen Ⅰ (COL Ⅰ) and collagen Ⅲ (COL Ⅲ) levels in HUVECs (Figure 2a-d). Consistently, high glucose augmented cells migration (Figure 2e). The results represented high glucose induced EndMT in HUVECs. The treatment of mannitol had no effect on cells migration, as well as CD31, αSMA and vimentin levels (Figure 2a-e). Previous study found ENO1 is involved in hyperglycemia-induced EMT. So, we measured ENO1 levels, and found high glucose increased ENO1 levels in HUVECs (Figure 2f, g). To further determine whether ENO1 takes a crucial part in high glucose-mediated EndMT, two independent si-ENO1 were adopted. The efficiency of si-ENO1 was confirmed in protein (Figure 2h) and mRNA (Figure 2i) levels. Our data showed si-ENO1 reversed high glucose-mediated reduction of CD31 levels and augment of vimentin, αSMA, COL Ⅰ and COL Ⅲ levels in HUVECs (Figure 2h, j-l). Meanwhile, si-ENO1 treatment inhibited high glucose-induced cells migration (Figure 2m). Our results represented that ENO1 played a positive role in regulation of EndMT in HUVECs cultured in high glucose condition.

### KMT5A reduction was involved in high glucose-mediated EndMT via increase of ENO1 levels in HUVECs

**Figure 3:**
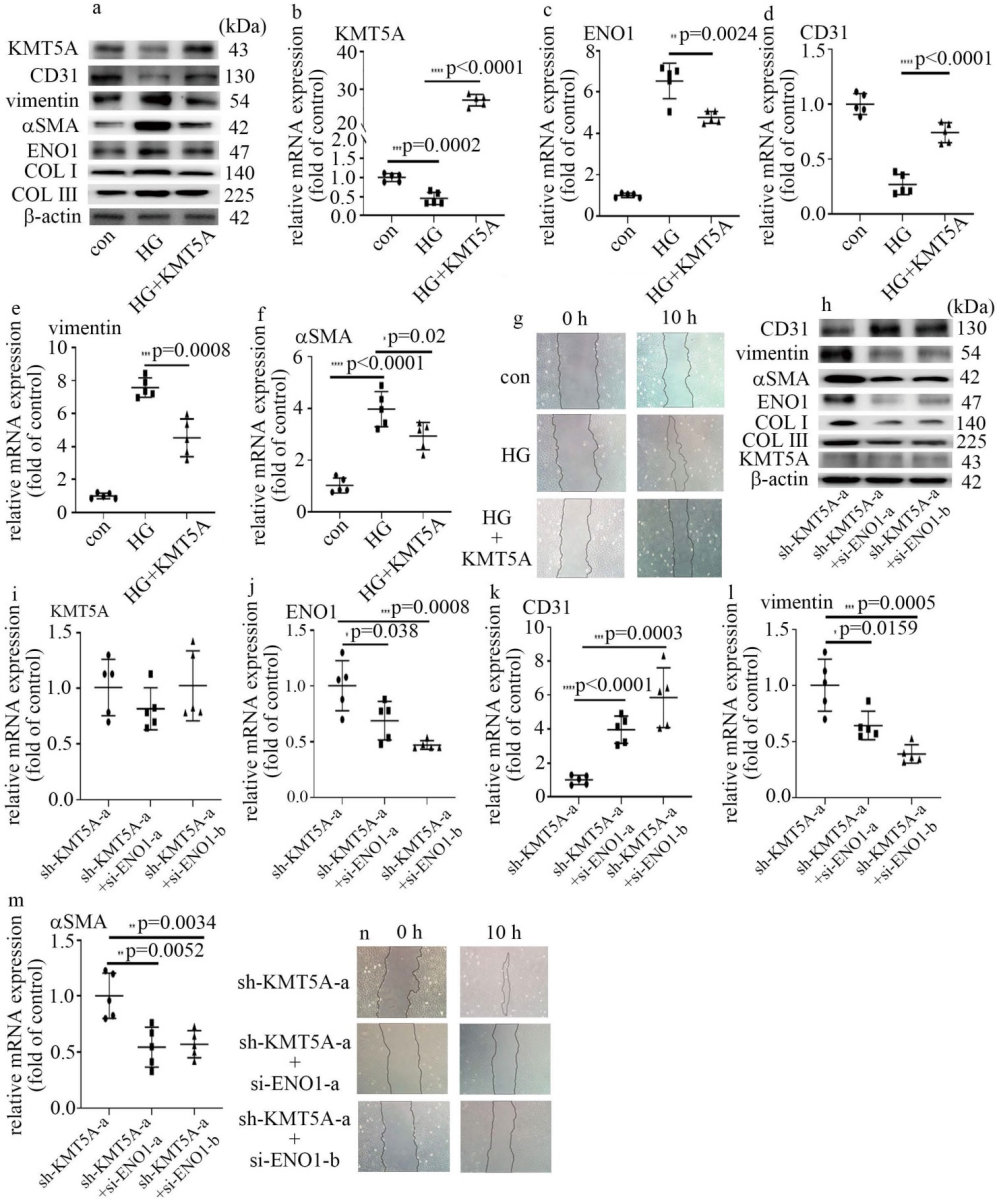
** KMT5A reduction was involved in high glucose-mediated EndMT via increase of ENO1 levels in HUVECs. (a)** Protein levels of KMT5A, CD31, vimentin, αSMA, ENO1, COLI and COLIII were tested by western blot in HUVECs with corresponding treatment. **(b)** mRNA levels of KMT5A were tested by qPCR with corresponding treatment. **(c)** mRNA levels of ENO1 were tested by qPCR with corresponding treatment. **(d)** mRNA levels of CD31 were tested by qPCR with corresponding treatment. **(e)** mRNA levels of vimentin were tested by qPCR with corresponding treatment. **(f)** mRNA levels of αSMA were tested by qPCR with corresponding treatment. **(g)** Cell migration was detected by scratch test. **(h)** Protein levels of KMT5A, CD31, vimentin, αSMA, ENO1, COLI and COLIII were tested by western blot in HUVECs with corresponding treatment. **(i)** mRNA levels of KMT5A were tested by qPCR with corresponding treatment. **(j)** mRNA levels of ENO1 were tested by qPCR with corresponding treatment. **(k)** mRNA levels of CD31 were tested by qPCR with corresponding treatment. **(l)** mRNA levels of vimentin were tested by qPCR with corresponding treatment. **(m)** mRNA levels of αSMA were tested by qPCR with corresponding treatment. **(n)** Cell migration was detected by scratch test. (* p<0.05, ** p<0.01, *** p<0.001, **** p<0.0001, n=5/group).

Our previous study represented that KMT5A plays a crucial role in high glucose-mediated endothelial damage 19-23. Moreover, KMT5A was reported to be involved in EMT 24. In this study, the protein and mRNA levels of KMT5A were decreased by high glucose treatment (Figure S2a, b). Consistently, the levels of H4K20me1, a direct target of KMT5A, decreased in HUVECs cultured in high glucose condition (Figure S2a). To explore whether KMT5A participated in the modulation of high glucose-induced ENO1 levels and EndMT, both loss-of-function and gain-of-function approaches were adopted in this study. The efficiency of KMT5A overexpression was confirmed in protein (Figure 3a) and mRNA (Figure 3b) levels. Our results represented that KMT5A upregulation counteracted high glucose-mediated increase of ENO1 levels (Figure 3a, c). Moreover, KMT5A upregulation reversed high glucose-induced decrease of CD31 expression (Figure 3a, d) and increase of vimentin, αSMA, COL Ⅰ and COLIII expression (Figure 3a, e, f), as well as cell migration (Figure 3g).

The efficiency of sh-KMT5A was confirmed by western blot (Figure S2c) and qPCR (Figure S2d). The impact of KMT5A silencing was similar to that of high glucose treatment (Figure S2c, e-i). To probe whether the effects of KMT5A silencing were obtained via increase of ENO1 levels, we used si-ENO1 in cells that KMT5A was down-regulated. Our results represented ENO1 downregulation reversed KMT5A silencing-induced EndMT in HUVECs (Figure 3h-n). Our data illustrated that KMT5A reduction increased ENO1 levels in HUVECs cultured in high glucose condition, thus taking part in high glucose-induced EndMT.

### KMT5A interacted with RFX1

**Figure 4:**
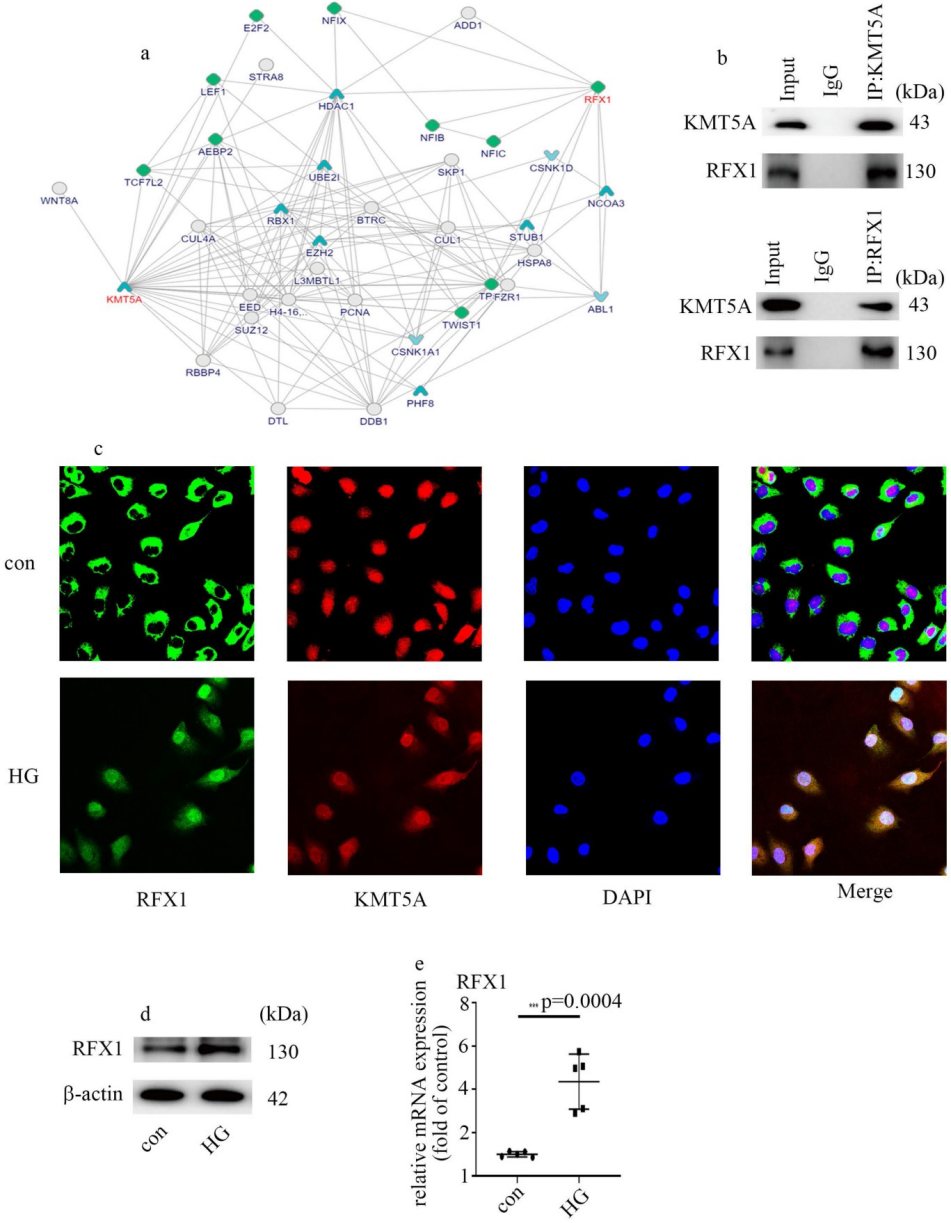
** KMT5A interacted with RFX1. (a)** A mount of molecules which interacted with KMT5A. **(b)** The interaction between KMT5A and RFX1 was proved by co-IP in HUVECs. **(c)** The colocalization of KMT5A and RFX1 in HUVECs. **(d)** Protein levels of RFX1 were tested by western blot in HUVECs with corresponding treatment. **(e)** mRNA levels of RFX1 were tested by qPCR with corresponding treatment. (* p<0.05, ** p<0.01, *** p<0.001, **** p<0.0001, n=5/group).

To explore the underlying mechanism by which KMT5A modulates ENO1 expression and EndMT, we analyzed the potential molecules which interact with KMT5A via bioinformatics. A mount of molecules which interacted with KMT5A were listed in Figure 4a (https://inbio-discover.intomics.com/map.html#search). Previous study indicated that RFX1 is an inducer of EMT 25. The interaction between KMT5A and RFX1 was verified by Co-IP in HUVECs (Figure 4b). Double immunofluorescent staining discovered that KMT5A colocalized with RFX1 in HUVECs (Figure 4c). Moreover, our data also represented that high glucose mediated RFX1 translocation from cytoplasm to nucleus (Figure 4c). Further, high glucose was found to augment RFX1 levels (Figure 4d, e). The present study indicated high glucose treatment modulated the cellular localization of RFX1, as well as RFX1 levels in HUVECs.

### RFX1 participated in high glucose-induced EndMT via augment of ENO1 levels in HUVECs

**Figure 5:**
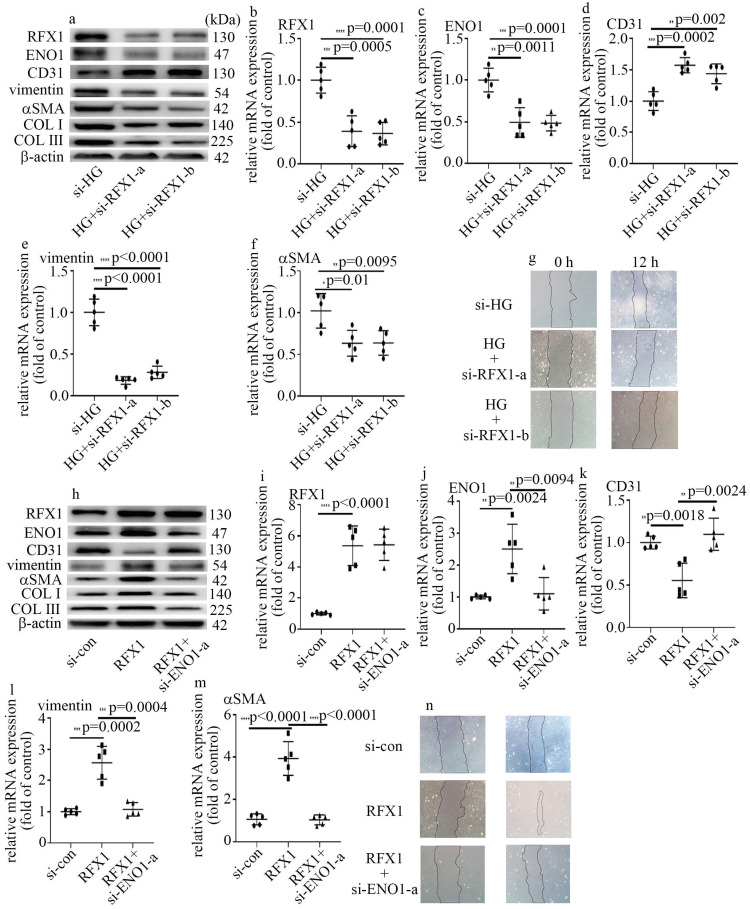
** RFX1 participated in high glucose-induced EndMT via augment of ENO1 expression in HUVECs. (a)** Protein levels of RFX1, CD31, vimentin, αSMA, ENO1, COLI and COLIII were tested by western blot in HUVECs with corresponding treatment. **(b)** mRNA levels of RFX1 were tested by qPCR with corresponding treatment. **(c)** mRNA levels of ENO1 were tested by qPCR with corresponding treatment. **(d)** mRNA levels of CD31 were tested by qPCR with corresponding treatment. **(e)** mRNA levels of vimentin were tested by qPCR with corresponding treatment. **(f)** mRNA levels of αSMA were tested by qPCR with corresponding treatment. **(g)** Cell migration was detected by scratch test. **(h)** Protein levels of RFX1, CD31, vimentin, αSMA, ENO1, COLI and COLIII were tested by western blot in HUVECs with corresponding treatment. **(i)** mRNA levels of RFX1 were tested by qPCR with corresponding treatment. **(j)** mRNA levels of ENO1 were tested by qPCR with corresponding treatment. **(k)** mRNA levels of CD31 were tested by qPCR with corresponding treatment.** (l)** mRNA levels of vimentin were tested by qPCR with corresponding treatment. **(m)** mRNA levels of αSMA were tested by qPCR with corresponding treatment. **(n)** Cell migration was detected by scratch test. (* p<0.05, ** p<0.01, *** p<0.001, **** p<0.0001, n=5/group).

To probe whether RFX1 participated in the modulation of high glucose-mediated increase of ENO1 levels and EndMT, we employed both loss-of-function and gain-of-function approaches in this study. The efficiency of si-RFX1 was exhibited via western blot (Figure 5a) and qPCR (Figure 5b). RFX1 silencing was found to counteract high glucose-mediated increase of ENO1 levels (Figure 5a, c). Moreover, si-RFX1 reversed high glucose-induced decrease of CD31 levels (Figure 5a, d) and increase of vimentin, αSMA, COLI and COLIII levels (Figure 5a, e, f), as well as cell migration (Figure 5g). Further, the impact of RFX1 upregulation was similar to that of the high glucose treatment (Figure 5h-n). To verify whether the impact of RFX1 upregulation was obtained via increase of ENO1 expression, we downregulation of ENO1 in RFX1-overexpressed cells. The present study indicated that ENO1 downregulation reversed RFX1 upregulation-mediated decrease of CD31 levels (Figure 5h, k) and increase of vimentin, αSMA, COL Ⅰ and COLIII levels (Figure 5h, l, m), as well as augment of cell migration (Figure 5n) in HUVECs. The present study indicated that RFX1 upregulation increased ENO1 expression in high glucose-treated cells, thus contributing to high glucose-induced EndMT.

### KMT5A associated with RFX1 to modulate ENO1 transcript in HUVECs

**Figure 6:**
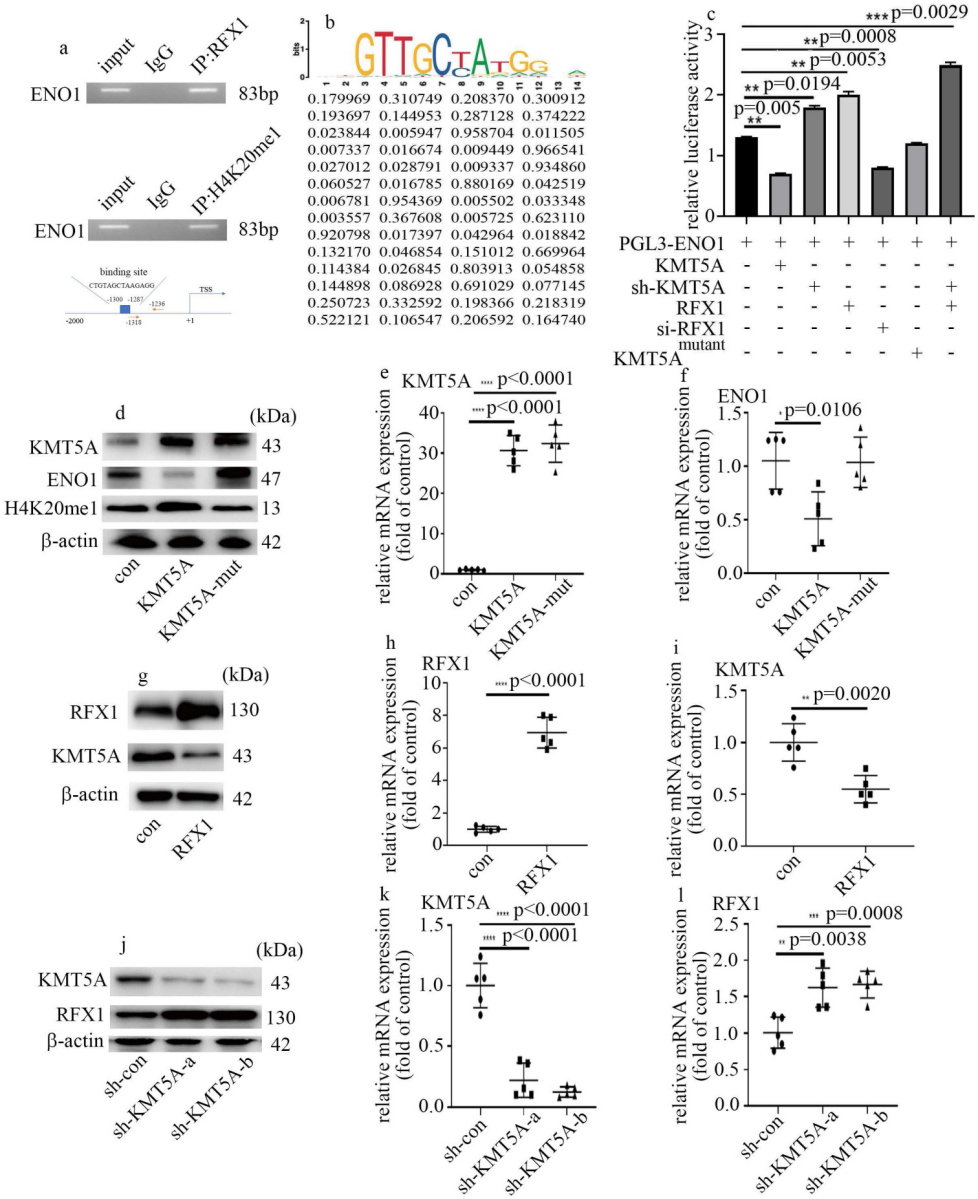
** KMT5A associated with RFX1 to modulate ENO1 transcript in HUVECs. (a)** RFX1 and H4K20me1 positioned on the promoter region of ENO1. **(b)** The predicted RFX1 binding site on ENO1 promotor region. **(c)** The activity of ENO1 promoter was detected with corresponding treatment. **(d)** Protein levels of KMT5A, ENO1 and H4K20me1 were tested by western blot in HUVECs with corresponding treatment. KMT5A-mut means mutant KMT5A^R259G^. **(e)** mRNA levels of KMT5A were tested by qPCR with corresponding treatment. **(f)** mRNA levels of ENO1 were tested by qPCR with corresponding treatment. **(g)** Protein levels of KMT5A and RFX1 were tested by western blot in HUVECs with corresponding treatment. **(h)** mRNA levels of RFX1 were tested by qPCR with corresponding treatment. **(i)** mRNA levels of KMT5A were tested by qPCR with corresponding treatment. **(j)** Protein levels of KMT5A and RFX1 were tested by western blot in HUVECs with corresponding treatment. **(k)** mRNA levels of KMT5A were tested by qPCR with corresponding treatment. **(l)** mRNA levels of RFX1 were tested by qPCR with corresponding treatment. (* p<0.05, ** p<0.01, *** p<0.001, **** p<0.0001, n=5/group).

Next, to determine whether ENO1 is directly modulated by KMT5A and RFX1, ChIP assay was used to detect the genome-wide localization of H4K20me1 and RFX1 in HUVECs cultured in normal glucose condition. H4K20me1 and RFX1 were both found to position on the promoter region of ENO1 (Figure 6a). The predicted RFX1 binding site and the primer positions are exhibited in figure 6a. The motif logo and position weight matrix (http://jaspar.genereg.net/) were exhibited in the up and down panel, accordingly (Figure 6b). Moreover, KMT5A upregulation and RFX1 silencing both reduced the activity of ENO1 promoter (Figure 6c). KMT5A silencing not only augmented the activity of ENO1 promoter but also enhanced the positive effect of RFX1 on the activity of ENO1 promoter (Figure 6c). These results represented KMT5A associated with RFX1 to regulate ENO1 transcript. Further, KMT5A upregulation attenuated ENO1 levels, while mutant KMT5A^R259G^ (dominant-negative role in H4K20 monomethylation 22) had no effect on ENO1 expression (Figure 6d-f). These results demonstrated that KMT5A-modulated H4K20me1 was necessary to modulate ENO1 transcript in HUVECs. Furthermore, RFX1 upregulation inhibited KMT5A levels (Figure 6g-i). Consistently, sh-KMT5A increased RFX1 levels (Figure 6j-l). These results represented that KMT5A and RFX1 suppressed each other in HUVECs.

### Hyperglycemia-mediated reduction of KMT5A and augment of RFX1 were confirmed in DN patients and rats

**Figure 7:**
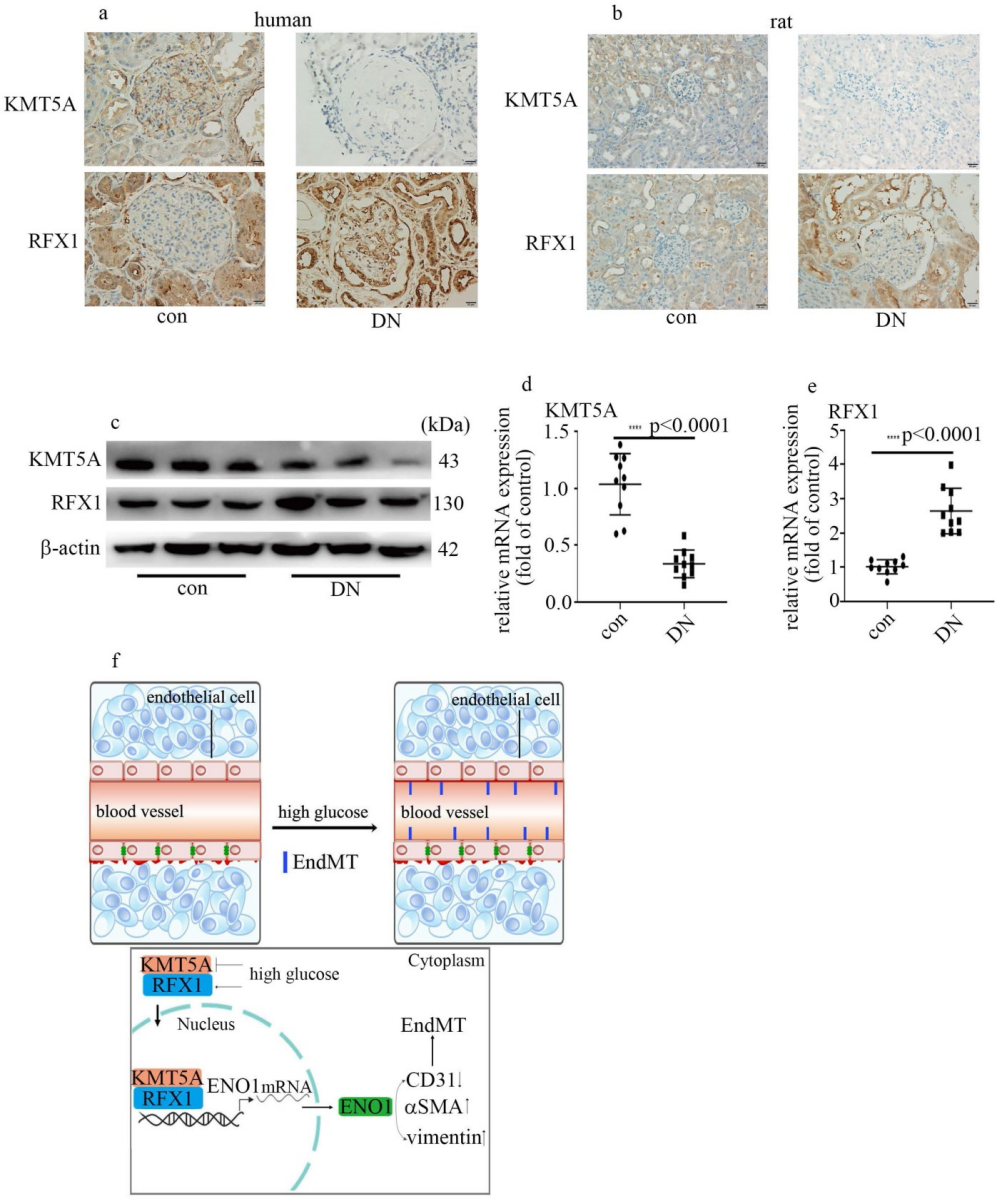
** Hyperglycemia-mediated reduction of KMT5A and augment of RFX1 were confirmed in DN patients and rats. (a)** IHC results of KMT5A and RFX1 in kidney tissue of control and DN participates (n = 20/group). Magnification: ×20. Scale bar: 20 μM. **(b)** IHC results of KMT5A and RFX1 in kidney tissue of control and DN rats (n = 10/group). Magnification: ×20. Scale bar: 20 μM. **(c)** Protein levels of KMT5A and RFX1 in kidney tissue of control and DN rats (n = 10/group). **(d)** mRNA levels of KMT5A were tested by qPCR in kidney tissue of control and DN rats (n = 10/group). **(e)** mRNA levels of RFX1 were tested by qPCR in kidney tissue of control and DN rats (n = 10/group). **(f)** Schematic representation of the working model. (* p<0.05, ** p<0.01, *** p<0.001, **** p<0.0001).

To probe if the levels of KMT5A and RFX1 in *in vivo* study were similar to those in *in vitro* study, we measured KMT5A and RFX1 levels in kidney tissue and/or aorta tissues of DN participants and rats. Our data represented KMT5A reduced, as well as RFX1 augmented in kidney tissue and/or aorta tissues of DN participants and rats (Figure 7a-e; Figure S3). In summary, these data represented that KMT5A and RFX1 associated to regulate ENO1 transcript, thus taking part in EndMT in DN participants and rats (Figure 7f).

## Discussion

The present study demonstrated that hyperglycemia, via upregulation of ENO1 levels, participated in EndMT and vascular endothelial cells damage, thus mediating the genesis and development of DN. Moreover, high glucose mediated inhibition of KMT5A expression and increase of RFX1 expression. Further, RFX1 and H4K20me1 was proved to position on the promoter region of ENO1. Mechanistic studies illustrated KMT5A played synergy with RFX1 to participate in modulation of ENO1 expression, thus inducing EndMT in HUVECs cultured in high glucose condition.

EMT, serving as an intricate cell phenotypic reconstruction, takes an important part in organ damage 26. It was reported that EMT of renal tubular epithelial cells exhibits an important role in the development of kidney fibrosis 27. Lately, EndMT in glomeruli was reported to take an important part in the genesis and development of DN 7, 8. Our data represented that CD31 decreased, and vimentin and αSMA increased in the glomeruli of DN patients and rats (Figure 1a, b). Our results were quite similar to a recent study which represented EndMT was involved in genesis and development of DN 28. To verify hyperglycemia played a crucial role in EndMT in DN patients and rats, we used HUVECs cultured in high glucose condition as an *in vitro* model. Western blot and qPCR results of CD31, vimentin and αSMA indicated that high glucose was involved in the genesis and development of EndMT, which was consistent with a previous study 29. It was deduced that EMT and EndMT may have corporate regulators 13. ENO1, which is important in regulation of aerobic glycolysis, was also reported to participate in hyperglycemia-induced EMT 16. In this study, the expression of ENO1 augmented in DN participants and rats (Figure 1). Moreover, high glucose enhanced ENO1 levels (Figure 2f, g) and mediated EndMT (Figure 2a-e) in HUVECs. Silencing of ENO1 levels counteracted high glucose-induced EndMT (Figure 2h-m). These data represented that ENO1 participated in high glucose-induced EndMT.

KMT5A was indicated to modulate angiogenesis in HUVECs 18. Our previous studies illustrated that KMT5A takes an important part in high glucose-mediated endothelial inflammation 19, 20, 22, 23 and antioxidant imbalance 21 in endothelial cells, thus participating in vascular endothelial damage 19-23. However, the mechanism by which KMT5A modulated EndMT in endothelial cells has not been well studied. Previous research represented KMT5A participates in EMT in some situations 24, 30. As a part of EMT, the modulators of EMT may also take a crucial part in modulation of EndMT 13. Our results represented that KMT5A upregulation reversed high glucose-mediated increase of ENO1 levels (Figure 3a, c) and augment of EndMT (Figure 3a-g). Moreover, H4K20me1 was positioned on the promoter region of ENO1 (Figure 6a). Further, ENO1 silencing reversed sh-KMT5A-mediated augment of EndMT (3h-n). Our results represented that high glucose-induced decrease of KMT5A levels was involved in EndMT via elevation of ENO1 levels in HUVECs cultured in high glucose condition.

RFX1 serves as a vital transcription factor, which participates in various biologic processes, including hearing 31, innate immune response 32, inflammatory response 33 and gene transcriptional repression or activation 34-36. Recently, RFX1 was proved to modulate EMT in tumor cells 25. In this study, our data represented that RFX1 levels elevated in HUVECs cultured in high glucose condition (Figure 4d, e). Moreover, RFX1 silencing counteracted high glucose-mediated increase of ENO1 and occurrence of EndMT (Figure 5a-g). Further, RFX1 overexpression elevated ENO1 levels and induced EndMT, which was reversed by ENO1 silencing (Figure 5h-n). The present study represented that RFX1 participated in high glucose-mediated occurrence of EndMT via increase of ENO1 levels in HUVECs cultured in high glucose condition.

It was demonstrated that the transcriptional activity of RFX1 is modulated by epigenetic modifications 32. Our data illustrated that KMT5A interacted with RFX1 (Figure 4b, c). Moreover, the promoter region of ENO1 was both positioned by RFX1 and H4K20me1 (Figure 6a). Further, KMT5A silencing increased the positive action of RFX1 on the activity of ENO1 promoter (Figure 6c). The results represented that KMT5A played synergy with RFX1 to modulate ENO1 transcript, thus inducing EndMT in HUVECs cultured in high glucose condition (Figure 7f). Further, KMT5A overexpression decreased ENO1 expression. However, mutant KMT5A^R259G^ did not affect ENO1 levels (Figure 6d-f). The present study represented that KMT5A-modulated H4K20me1 played a crucial role in the modulation of ENO1 levels.

The present study has some limitations. First, whether KMT5A interacted to RFX1 in a direct or indirect way needs our further confirmation. Second, other primary endothelial cell needs to be employed to confirm the present study, as that only HUVECs was used to build cellular model in this study was insufficient. Third, the mechanism that KMT5A and RFX1 suppressed each other needs our deep exploration. Fourth, the underlying mechanism that ENO1 induced EndMT in HUVECs cultured in high glucose condition needs our further study.

In summary, the present study represented that KMT5A expression decreased, RFX1 and ENO1 expression increased, and EndMT was mediated in glomeruli of DN patients and rats. Our data also illustrated that high glucose mediated EndMT via enhancement of ENO1 levels in HUVECs cultured in high glucose condition. Moreover, high glucose inhibited KMT5A expression and upregulated RFX1 expression. Further, KMT5A played synergy with RFX1 to adjust ENO1 transcript, thus contributing to high glucose-induced EndMT in HUVECs cultured in high glucose condition.

## Materials and Methods

### Human specimens

Twenty kidney carcinoma patients (nondiabetics with normal renal function) were enrolled in the present study as control participants. Twenty biopsy-diagnosed DN patients were recruited as DN participates in this study. This research was ratified by the Ethics Committee of Huzhou Central Hospital (Ethics Number: 20191209-01) and in accordance with the Declaration of Helsinki. All the participants signed informed consent forms in this study.

### Construction of Rat DN model

4-weeks-old male Sprague Dawley rats, obtained from Shanghai SLAC Laboratories, were randomly allocated to corresponding treatment groups. This research was in accordance with the Guide for the Care and Use of Laboratory Animals of Fudan University Shanghai Cancer Center. Animals were randomly divided into the control group (con, n=10) and the DN group (DN, n=10). The control animals were intraperitoneally received with citrate buffer (0.1 M, pH 4.5) only once. The DN animals, raised by a high sugar-fat diet for 2 weeks, were intraperitoneally received with 50 mg/kg streptozotocin only once. Blood glucose was measured 3 days after streptozotocin treatment. After a standard laboratory chow for 6 weeks, the animals were euthanized with the use of intraperitoneal administration of 40 mg/kg thiopental sodium.

### Harvest of animal blood and urine samples

The day before euthanasia, 24 hours urine was gathered with the use of metabolic cages. Blood specimens were gathered in EDTA vacutainer tubes. The samples were kept frozen at -80°C until analysis. A creatinine assay kit (Jiancheng Bio, Nanjing, China) and a BUN assay kit (Jiancheng Bio, Nanjing, China) was used to detect serum creatinine and urea nitrogen (BUN) correspondingly. An enhanced BCA protein assay kit (Beyotime, Shanghai, China) was used to detect urinary albumin.

### Immunohistochemistry (IHC)

IHC was used to measure protein expression of the specimens of participants and animals with the specific primary antibody at 4℃ overnight in a humidified chamber. The primary antibody included anti-KMT5A (ProteinTech, Wuhan, China), anti-ENO1 (Proteintech, Wuhan, China), anti-regulatory factor X1 (RFX1, Santa Cruz Biotechnology, Santa Cruz, CA), anti-vimentin (Proteintech, Wuhan, China), anti-CD31 (Proteintech, Wuhan, China) and anti-αSMA (Cell Signaling Technology, Danvers, MA) antibodies.

### Cell culture and reagents

HUVECs were acquired from American Type Culture Collection (ATCC; Manassas, USA). Cells were cultured in 5 mM glucose Dulbecco's modified Eagle medium (DMEM, HyClone Laboratories, Logan, USA) with 10% fetal bovine serum and 1% penicillin/streptomycin (100g/ml) 6 days as control group. To simulate hyperglycemia-mediated vascular endothelial damage, HUVECs were cultured in 25 mM glucose DMEM (HyClone Laboratories, Logan, USA) with 10% fetal bovine serum and 1% penicillin/streptomycin (100 g/ml) 6 days as high glucose treatment group (HG). The osmotic control was composed with 5 mM glucose and 20 mM mannitol.

### Western blot analysis

Whole-cell extracts were acquired via protein lysis buffer (Cell Signaling Technology, Danvers, MA). Same amounts of proteins (60μg) of each group were segregated by SDS-PAGE and moved to PVDF membranes (Millipore, Billerica, USA). After blocked by 5% fat-free milk at room temperature for 1 hour, the PVDF membranes were immersed in specific primary antibodies at 4 °C overnight. The primary antibodies included anti-β-actin (ProteinTech, Wuhan, China), anti-KMT5A (ProteinTech, Wuhan, China), anti-RFX1 (Santa Cruz Biotechnology, Santa Cruz, CA), anti-ENO1 (ProteinTech, Wuhan, China), anti-CD31 (ProteinTech, Wuhan, China), anti-H4K20me1 (Abcam, Cambridge, UK), anti-vimentin (ProteinTech, Wuhan, China), anti-αSMA (Cell Signaling Technology, Danvers, MA), anti-COL Ⅰ (Abclonal, Wuhan, China) and anti-COL Ⅲ (Abclonal, Wuhan, China) antibody. Then, corresponding secondary antibodies were used to immerse the PVDF membranes at room temperature for 1 hour. Then, the PVDF membranes were washed with PBST 3 times. Subsequently, the protein signal was displayed by an ECL system.

### Quantitative real-time PCR (qPCR)

In the present study, TRIzol (Invitrogen, Grand Island, NY, USA) was adopted to obtain total RNA. Complementary DNA (cDNA) was prepared by PrimeScript RT reagent (TaKaRa Bio, Dalian, China). qPCR was implemented with the use of Hieff UNICON® qPCR TaqMan Probe Master Mix (Yeasen, Shanghai, China) on an ABI7500 Real-Time PCR system (Applied Biosystems). The primers were exhibited in Table S1.

### Scratch assay

HUVECs (1×10^5^) were seeded into the 6-well plate, and five biological replicates were observed in each group. When the cells achieved a confluence of 95%, 10 μl pipette was used to scratch the bottom of each well. After cells washed three times with PBS, a serum-free culture medium was added. Ten hours after the capture of the first photograph of the scratches (0 hour), the second photograph of the scratches was acquired.

### Co-immunoprecipitation (CoIP)

Whole-cell protein lysates were acquired via protein lysis buffer containing PMSF (Beyotime Biotechnology, Shanghai), and 20μl of the lysates was gathered as an input. For endogenous immunoprecipitation, the surplus samples were mixed with anti-KMT5A antibody (ProteinTech, Wuhan, China), anti-RFX1 antibody (Santa Cruz Biotechnology, Santa Cruz, CA), or IgG, supplemented with 50 μl protein A/G Dynabeads (Thermo Fisher, USA) at 4 °C overnight. Finally, 10 μl of input, IgG and immunoprecipitation samples were applied to western blot analysis.

### Immunofluorescence (IF) staining

Cells were seeded onto the glass slides. Paraformaldehyde (4%) and Triton X-100 (0.3%) were used to fix and permeabilize the cells respectively. After blocked, HUVECs were immersed with the specific primary antibodies at 4℃ overnight. 4,6-diamidino-2-phenylindole (DAPI) was used to stain the nucleus in HUVECs. The images were displayed with a confocal Leica fluorescence microscope.

### siRNA, shRNA and KMT5A mutant treatments

Cells were transfected with si-ENO1, sh-KMT5A, KMT5A plasmid, RFX1 plasmid, si-RFX1 and mutant KMT5A^R295G^ plasmid (dominant-negative role in H4K20 monomethylation) 22 with the use of Lipofectamine 3000 (Invitrogen, USA). The sequences of sh-KMT5A (Biotend, Shanghai, China) were sh-KMT5A-a, 5'-CAACAGAATCGCAAACTTA-3', and sh-KMT5A-b, 5'-CAACAGAATCGCAAACTTA-3'. The sequences of si-ENO1 (Biotend, Shanghai, China) were si-ENO1-a, 5'- CGAGAUGGAUGGAACAGAAdTdT-3', and si-ENO1-b, 5'- GAGCAGAGGUUUACCACAAdTdT-3'. The sequences of si-RFX1 (Biotend, Shanghai, China) were si-RFX1-a, 5'-CGUGGUCACUGUCUCUGAAdTdT-3', and si-RFX1-b, 5'-CCAGCUGACCAACAUCCAAdTdT-3'.

### Chromatin immunoprecipitation (ChIP) assay

ChIP assays were implemented via ChIP Kit ab500 (Abcam, Cambridge, UK). Briefly, cells (1×10^7^) were immobilized by 1% formaldehyde. The reaction of cross-linking was ceased via glycine. A Microson XL ultrasonic cell disruptor XL (Misonix, New York) was employed to clip the chromatin. 10 μl sonicated samples were collected as input. The surplus samples were cultured with anti-RFX1 antibody (Santa Cruz Biotechnology, Santa Cruz, CA), anti-H4K20me1 antibody (Abcam, Cambridge, UK) or IgG at 4 °C overnight. The cross-linking between DNA and protein was ceased by culture at 65 °C for 2 hours. After being purified, the occupied DNA sequences were determined by PCR. The primer sequences of ENO1 were list as below: forward, 5'-AACGCCATGGAAACCTGTCT-3', and reverse, 5'-TCTGGTCAGCTGCAAAACCT-3'.

### Dual-luciferase Assay

The activity of ENO1 promoter was measured by a Dual-luciferase Reporter Assay System (Promega, Madison, United States). The promoter region of ENO1 (2000 bp upstream from the transcript start site) was amplified and ligated into the pGL3-basic vector to establish pGL3-ENO1. The pGL3-ENO1 plasmid was then transfected into the cells.

### Statistical analysis

The data were exhibited as mean ± standard deviation. The experiments were performed separately at least 5 times. n represented the times of biological replicates. Two-tailed unpaired t-tests or one-way ANOVAs were employed to compare the difference between the groups. P < 0.05 was considered significant.

## Supplementary Material

Supplementary figures and table.

## Figures and Tables

**Table 1:** Information of participants and animals in control (con) group and Diabetic Nephropathy (DN) group

Human			
**Variables**	**Con**	**DN**	**P-value**
Male (%)	45	50	0.76
Age (years)	53.6±9.7	53.6±9.5	0.91
BMI (kg/m^2^)	23.0±0.92	23.5±1.9	0.37
SBP (mmHg)	109.85±4.57	144.3±15.7	<0.0001
DBP (mmHg)	58.4±5.9	78.25±11.5	<0.0001
HbA1C (%)	5.16±0.48	8.1±1.8	<0.0001
FBG (mmol/l)	5.0±0.73	7.3±2.7	<0.0001
CREA (umol/l)	56.8±8.1	188.0±124.5	<0.0001
ALB (g/L)	48.5±3.7	30.1±8.1	<0.001
CCr (ml/min)	98.5±9.5	54.8±33.3	<0.0001
24hUTP (mg)	108.0±12.7	3759.1±3303.1	<0.0001
UA (umol/L)	211.2±36.8	393.8±73.3	<0.0001
TP (g/L)	67.6±8.3	55.6±8.1	<0.001
**Rats**			
**Variables**	**Con**	**DM**	**P-value**
FBG (mmol/l)	3.9±0.2	26.2±2.2	<0.0001
CREA (umol/l)	21.0±2.2	39.7±9.7	<0.0001
UREA (mmol/l)	2.3±0.2	6.4±1.1	<0.0001
24hUTP (mg)	31.7±4.5	357.9±70.3	<0.0001

Data are presented as means ± SD. BMI (Body Mass Index), SBP (systolic blood pressure), DBP (diastolic blood pressure), HbA1c (glycated hemoglobin), FBS (fasting blood sugar), CREA (creatinine), ALB (albumin), CCr (Creatnine Clearance), 24hUTP (24-hour urinary protein quantity), UA (Uric Acid), TP (Total Protein).

## Abbreviations

DN: Diabetic nephropathy
EndMT: endothelial-to-mesenchymal transition
ENO1: Enolase1
KMT5A: Lysine Methyltransferase 5A
RFX1: regulatory factor X1
H4K20me1: histone H4 lysine20 methylation
αSMA: α-smooth muscle actin
COL I: collagen I
COL III: collagen III
