# Supplementary material for: KMT5A downregulation participated in High Glucose-mediated EndMT via Upregulation of ENO1 Expression in Diabetic Nephropathy
Source: Int J Biol Sci. 2021 Oct 3;17(15):4093–107. doi: 10.7150/ijbs.62867 (PMC8579450; doi:10.7150/ijbs.62867)
Supplement: Supplementary file 1 — Supplementary figures and table. [file ijbsv17p4093s1.pdf]

Supplementary Figure Legends

Figure S1. Decrease of CD31 levels, augment of vimentin and αSMA expression, and increase of ENO1 expression in aorta tissues of DN rats

(a) IHC results of CD31, vimentin, αSMA and ENO1 in aorta tissues of control and DN rats (n = 10/group). Magnification: ×20. Scale bar: 20 μM. (b) Protein levels of CD31, vimentin, αSMA and ENO1 in aorta tissues of control and DN rats (n = 10/group). (c) mRNA levels of CD31 in aorta tissues of control and DN rats (n = 10/group). (d) mRNA levels of vimentin in aorta tissues of control and DN rats (n = 10/group). (e) mRNA levels of αSMA in aorta tissues of control and DN rats (n = 10/group). (f) mRNA levels of ENO1 in aorta tissues of control and DN rats (n = 10/group). (* $p<0.05$, ** $p<0.01$, *** $p<0.001$, **** $p<0.0001$)

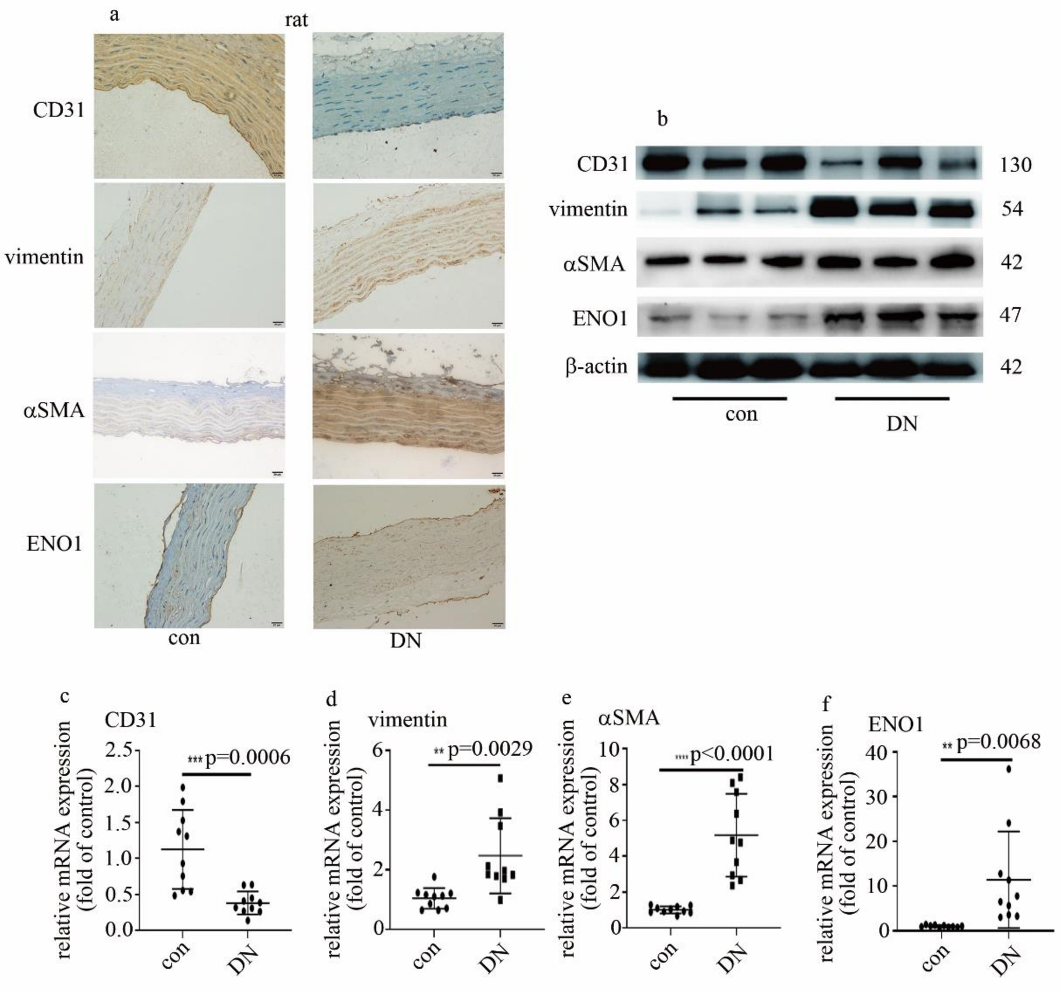

a
rat
CD31
vimentin
αSMA
ENO1
con
DN
b
CD31
vimentin
αSMA
ENO1
β-actin
130
54
42
47
42
con
DN
c
CD31
***p=0.0006
relative mRNA expression (fold of control)
con
DN
d
vimentin
**p=0.0029
relative mRNA expression (fold of control)
con
DN
e
αSMA
****p<0.0001
relative mRNA expression (fold of control)
con
DN
f
ENO1
**p=0.0068
relative mRNA expression (fold of control)
con
DN

Figure S2. sh-KMT5A mediated EndMT and increased ENO1 levels in HUVECs.

(a) Protein levels of KMT5A and H4k20me1 were tested by western blot in HUVECs with corresponding treatment. (b) mRNA levels of KMT5A were tested by qPCR with corresponding treatment. (c) Protein levels of KMT5A, CD31, vimentin, αSMA, ENO1, COL I and COL III were tested by western blot in HUVECs with corresponding treatment. (d) mRNA levels of KMT5A were tested by qPCR in HUVECs with corresponding treatment. (e) mRNA levels of ENO1 were tested by qPCR in HUVECs with corresponding treatment. (f) mRNA levels of CD31 were tested by qPCR in HUVECs with corresponding treatment. (g) mRNA levels of vimentin were tested by qPCR in HUVECs with corresponding treatment. (g) mRNA levels of αSMA were tested by qPCR in HUVECs with corresponding treatment. (i) Cell migration was detected by scratch test. (* $p<0.05$, ** $p<0.01$, *** $p<0.001$, **** $p<0.0001$, n=5/group)

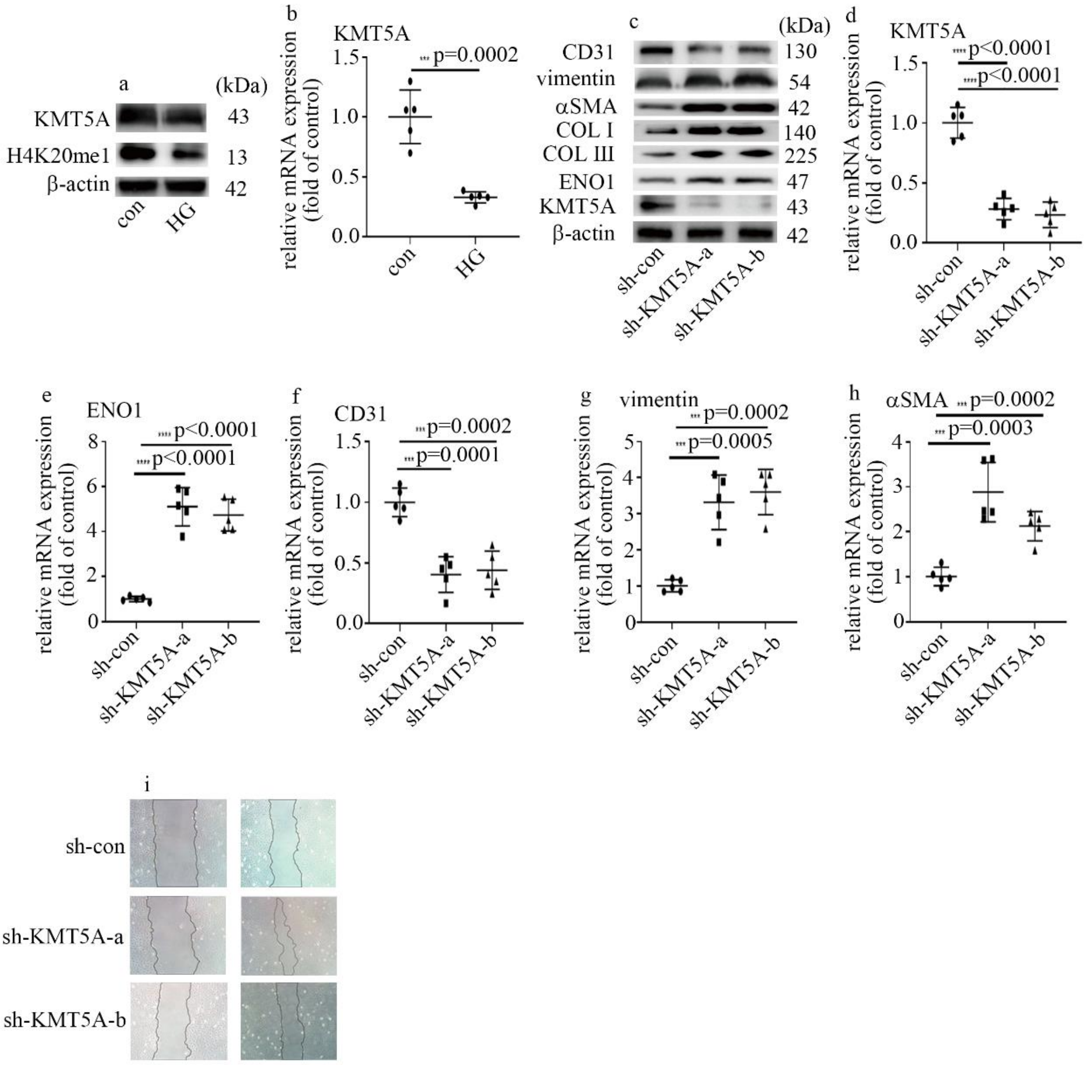
a
(kDa)
KMT5A 43
H4K20me1 13
β-actin 42
con
HG
b
KMT5A
p=0.0002
relative mRNA expression (fold of control)
1.5
1.0
0.5
0.0
con
HG
c
(kDa)
CD31 130
vimentin 54
αSMA 42
COL I 140
COL III 225
ENO1 47
KMT5A 43
β-actin 42
sh-con
sh-KMT5A-a
sh-KMT5A-b
d
KMT5A
p<0.0001
p<0.0001
relative mRNA expression (fold of control)
sh-con
sh-KMT5A-a
sh-KMT5A-b
e
ENO1
p<0.0001
p<0.0001
relative mRNA expression (fold of control)
sh-con
sh-KMT5A-a
sh-KMT5A-b
f
CD31
p=0.0002
p=0.0001
relative mRNA expression (fold of control)
sh-con
sh-KMT5A-a
sh-KMT5A-b
g
vimentin
p=0.0002
p=0.0005
relative mRNA expression (fold of control)
sh-con
sh-KMT5A-a
sh-KMT5A-b
h
αSMA
p=0.0002
p=0.0003
relative mRNA expression (fold of control)
sh-con
sh-KMT5A-a
sh-KMT5A-b
i
sh-con
sh-KMT5A-a
sh-KMT5A-b

Figure S3. Hyperglycemia-mediated decrease of KMT5A and increase of RFX1 were verified in DN rats.

(a) IHC results of KMT5A and RFX1 in aorta tissues of control and DN rats (n = 10/group). Magnification: ×20. Scale bar: 20 μM. (b) Protein levels of KMT5A and RFX1 in aorta tissues of control and DN rats (n = 10/group). (c) mRNA levels of KMT5A were tested by qPCR in aorta tissues of control and DN rats (n = 10/group). (d) mRNA levels of RFX1 were tested by qPCR in aorta tissues of control and DN rats (n = 10/group). (* $p<0.05$, ** $p<0.01$, *** $p<0.001$, **** $p<0.0001$)

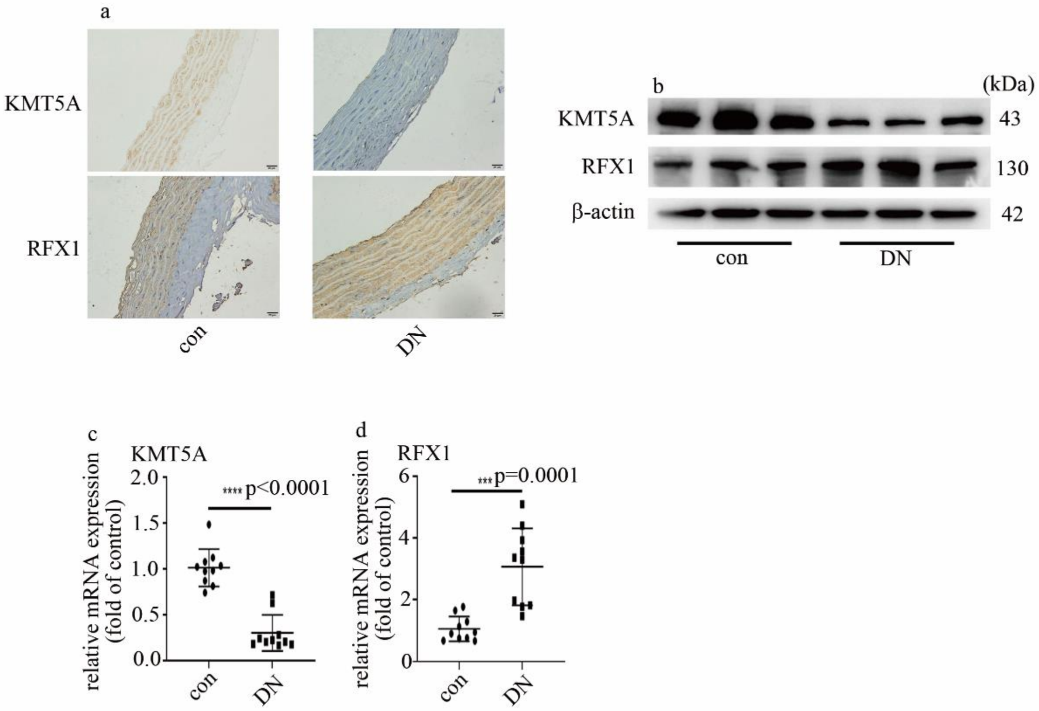
a
KMT5A
RFX1
con
DN
b
(kDa)
KMT5A
43
RFX1
130
β-actin
42
con
DN
c
KMT5A
relative mRNA expression (fold of control)
2.0
1.5
1.0
0.5
0.0
**** p<0.0001
con
DN
d
RFX1
relative mRNA expression (fold of control)
6
4
2
0
*** p=0.0001
con
DN

Table S1. Primers adopted for qPCR analysis.

species	RNA sequence
Human	
β-actin	F 5'- CGGCTACAGCTTCACCACCAC -3'
	R 5'- GCCATCTCTTGCTCGAAGTCCAG -3'
KMT5A	F 5'- TCCAGCAATCCTCCTCCTTCCTC -3'
	R 5'- CCAGCCTAAGCAACAGATCCAGA -3'
RFX1	F 5'- ATTCCCAAGCACAGATGGGG -3'
	R 5'- CCCCCTTCCCCATAGACAGA -3'
ENO1	F 5'- AGGGTGAGGGTTCTCCTCTG -3'
	R 5'- ACATGAGCAAAACGGGGACA -3'
VIMENTIN	F 5'- TACACAATTGCCTCTCCCCC -3'
	R 5'- ACTCCTGTCTGAGATTACCCT -3'
CD31	F 5'- ACAGGACCGCGTTTTATCCTT -3'
	R 5'- CCTTCCCAGTTCTGGGTTCTT -3'
αSMA	F 5'-ACCCAGCACCATGAAGATCA-3'
	R 5'- TTTGCGGTGGACAATGGAAG -3'
Rat	
β-actin	F 5'- CTTCCAGCCTTCCTTCCTGG -3'
	R 5'- GAGCCACCAATCCACACAGA -3'
KMT5A	F 5'- GCAGGAAGAGAACTCCGTCG -3'
	R 5'- AGAATCACATGACGGGGGTG -3'
RFX1	F 5'- GCACTGCCCCAGCTTTTTAC -3'
	R 5'- TGGCAACTGGCATCATCTGT -3'
ENO1	F 5'- AGGGTGGATTCGCACCTAAC -3'
	R 5'- GGTCAATTACCAGCACACGG -3'
CD31	F 5'- AGGCTGCCCTCAAACTCATC -3'
	R 5'- CCCAACACGGATGCAAAAGG -3'
VIMENTIN	F 5'- GCCCCACCCTGAACCTAAAC-3'
	R 5'- TGCCCTTCCCAACAATCACA-3'
αSMA	F 5'- CATCATGCGTCTGGACTTGG -3'
	R 5'- CCAGGGAAGAAGAGGAAGCA-3'
